# OP80 Generating Patient Preference Evidence For Health Technology Assessment: A Sustainable Roadmap

**DOI:** 10.1017/S0266462324001375

**Published:** 2025-01-07

**Authors:** Simon Fifer, Maya Joshi, Brittany Keen

## Abstract

**Introduction:**

Research with members of health technology assessment (HTA) bodies has uncovered key barriers to integrating patient preference (PP) data into HTA, including concerns about resources/time constraints and a lack of clarity around who is responsible for data generation. We sought to develop a roadmap that addresses these issues, outlining the roles and responsibilities of different stakeholders to foster more sustainable PP data generation.

**Methods:**

Based on a forthcoming article to be published in *The Patient*, this roadmap consists of a step-by-step approach for PP evidence generation. Real-world case studies and literature will be used to illustrate each stage, from identifying priority treatment areas and evidence gaps, forming a steering committee and engaging HTA members, to securing syndicated funding and disseminating results with full transparency.

**Results:**

In contrast to standard approaches to data generation, this roadmap focuses on proactive data collection, collaborating with those who will ultimately use the data (HTA), and pooling resources to mitigate costs and the risk of bias. The roadmap can be applied to all preference-sensitive treatment areas and across health systems/countries. It is designed to be a continuous process, whereby preferences are regularly updated to align with changes to the treatment landscape. A graphic summary of the roadmap is available for viewing at this link: https://cappre.info/images/HTAprocess.pdf

**Conclusions:**

Patient preference data has the potential to make healthcare decision-making more informed, socially legitimate, transparent, and accountable to the patient community. However, current approaches to capturing PP data can be resource intensive with narrow applicability in their findings. The present roadmap offers an alternative, sustainable solution.

